# Safety assessment of L-Arg oral intake in healthy subjects: a systematic review of randomized control trials

**DOI:** 10.1007/s00726-023-03354-6

**Published:** 2023-11-10

**Authors:** Yui Kuramochi, Mai Murata, Akihide Sumino, Hideko Sone, Kohsuke Hayamizu

**Affiliations:** Laboratory of Food Chemistry, Yokohama University of Pharmacy, 601 Matano-Cho, Totsuka-Ku, Yokohama, Kanagawa 245-0066 Japan

**Keywords:** L-Arg, Systematic review, Safety, NOAEL, Gastrointestinal symptom, Weighted change-point regression model

## Abstract

L-Arg is a nonessential amino acid but has many physiological roles. Accordingly, L-Arg has been used in various fields, but there is only limited information available about its safety upon overdose. Generally, the no-observed adverse effect level (NOAEL) is used when setting the upper amount for chemical substances. Recently, systematic reviews have been used to assess the safety as well as the effectiveness and usefulness of them. Therefore, we conducted an assessment of the safety of the oral intake of L-Arg in healthy subjects using gastrointestinal symptoms as an index. We limited the study design to only double-blind randomized controlled trials and searched PubMed, Cochrane Library, EBSCOhost, and Ichushi-Web from inception until May 2021. Assessment of the quality of studies was conducted using the Cochrane Collaboration tool and Jadad score, and the random effects model was used for data analysis. Ultimately, 34 studies were selected for inclusion in this work. The dosage of L-Arg used in the studies ranged from 2000 to 30,000 mg/day (or/one-time dose), and the treatment duration was 1–84 days. The increased risk of gastrointestinal symptoms associated with L-Arg intake from 23 studies (647 participants in total) in which such symptoms were reported was 0.01 (95% confidence interval: – 0.02–0.04), which was not significant difference. NOAEL was estimated as 7531 mg/ one-time dose using a weighted change-point regression model (UMIN000046133).

*Registration and protocol*: Umin.ac.jp as UMIN000046133.

**Supplementary Information:**

The online version contains supplementary material available at 10.1007/s00726-023-03354-6.

## Introduction

Amino acids are used for the biosynthesis of proteins, such as metabolic enzymes and body structural proteins. Twenty different amino acids are used for the biosynthesis of proteins in the body, which are categorized into nine essential amino acids and eleven nonessential ones based on the nutritional necessity. Essential amino acids cannot be synthesized in the body, so they are used as parameters to assess the quality of proteins (amino acid score). Many different amino acids are now included in functional foods and supplements, as in general foods. However, there are no guidelines for the upper limit of safe ingestion of these amino acids.

L-Arg is a nonessential amino acid, but it plays many roles in the body. It is thought to be especially necessary for long-term growth and during development (Laidlaw and Kopple 1987). Accordingly, L-Arg is also called a “conditionally essential amino acid.” It is involved in protein synthesis as a proteinogenic amino acid and in the detoxification of ammonia as a constituent of the urea cycle. It also produces nitric oxide (NO) as a precursor of L-citrulline (L-Cit) (Shao and Hathcock 2008; Visek 1986). Moreover, it has been reported that NO promotes blood flow (Hambrecht et al. 2000) and improves male sexual function (Barassi et al. 2017), indicating NO’s vasodilatory activity. NO has also been reported to elevate growth hormone activity (Chromiak and Antonio 2002; Lind 2004; Visek 1986) and enhance wound healing (Cheshmeh et al. 2022; Lind 2004; Visek 1986). Consequently, NO is occasionally used for improving athletic performance (Shao and Hathcock 2008). For these reasons, L-Arg is often used as a dietary supplement, especially in sports nutrition, all over the world. L-Arg is also known for stimulating insulin secretion and is used as a non-glucose secretagogue (a drug that causes or stimulates secretion) to measure insulin secretion levels (Eremin O 1997). L-Arg is now being used in various fields for human health, but there is only limited information about its safety upon overdose.

Generally, the no-observed adverse effect level (NOAEL) is used as a parameter when setting the upper amount for chemical substances. NOAEL is determined via a test enabling judgment of the dose response, such as in vitro or in vivo study, an overdose test, or a tolerability test on humans. From the results of a nonclinical study using rats, Blachier et al. reported that the NOAEL of L-Arg is 3318 mg/kg BW/day in males and 3879 mg/kg BW/day in females (Blachier et al. 2021). The acceptable daily intake (ADI) for humans can be calculated using these data based on an uncertainty factor (UF) of 100 as follows:

$${\text{ADI }} = {\text{ UF}} \times {\text{NOAEL }} = { 1}/{1}0 \times {1}/{1}0 \times {3318 }\left( {{\text{mg}}/{\text{kg}}} \right) \, = { 3}.{318}\left( {{\text{mg}}/{\text{kg}}} \right)$$

This calculation is based on the body weight of adult men of 60 kg is 199.08 (mg/body/day). However, the mean intake of L-Arg from meals in adults is reported to be 59.71 mg/kg BW/day (Blachier et al. 2021). Thus, when the body weight is 60 kg, the corresponding intake is 3582.6 (mg/body/day). Trumbo et al. reported that the mean intake of dietary L-Arg is 4.2 g/day, while its 99th percentile is 10.1 g/day (Trumbo et al. 2002). In addition, Iguacel et al. reported that the mean intake is 3.6 g/day, while the interquartile range is 2.8–4.3 g/day (Iguacel et al. 2022). The estimated dietary intake level thus greatly surpasses the ADI and it is not realistic. This is not only the case for L-Arg, but also for other amino acids. For this reason, ADI calculated using NOAEL obtained from nonclinical trials cannot be used for assessing the safety of amino acids.

The NOAEL of L-Arg in healthy young people with a body weight is 70 kg has been estimated to be 20–40 g/day (Cynober et al. 2016). This value is estimated from the conversion table of the FDA using the dosage for which no toxic findings were obtained in a nonclinical trial in which rats or pigs were administered L-Arg over 90 days (Wu et al. 2016, Guidance for Industry 2005). Blachier et al. evaluated the safety of L-Arg using the NOAEL/UC ratio, based on the two parameters of NOAEL and usual consumption (UC) (Blachier et al. 2021). This ratio in humans and rats is 4.9 and 9.1, respectively, indicating that the range of safe use in humans is narrow compared with that in animals. The dose calculated from the conversion table is used as evidence for deciding on the initial amount such as medicine or food in a phase I study, which was administered to humans for the first time. Therefore, it isn't enough to decide this safe upper intake of L-Arg by only using the conversion table.

McNeal et al. conducted a double-blind placebo-controlled trial targeting overweight but otherwise healthy subjects aimed at clarifying the tolerability of L-Arg and reported that there were no problems associated with L-Arg at 30 g/day for 3 months (Cynober et al. 2016; McNeal et al. 2018). However, this test involved a wash-in period in which L-Arg was administered at 12 g/day for 1 week before group allocation and excluded subjects who dropped out during this period. We found that adverse events (AEs) had already occurred at 12 g/day because three subjects with intolerable gastrointestinal symptoms dropped out during the wash-in period. For this reason, the report actually focused on the results for subjects highly tolerant of L-Arg, so the generally safe amount of L-Arg remained unclear.

Blachier et al. (2021) and Elango (2023) published reviews on the NOAEL of L-Arg in humans. However, they also calculated the value by quoting the result of McNeal et al. (2018), so their NOAEL is probably lower than the reported value.

In recent years, systematic review (SR) has been used as an approach for assessing safety as well as effectiveness and usefulness of amino acids. However, to the best of our knowledge, no SR assessing the safety of L-Arg has been reported yet. For this reason, we designed a study to assess the safety of L-Arg using SR.

Hayamizu et al. (2019) used L-lysine (L-Lys) as a subject and considered the assessment of its safety by SR. They provisionally reported its NOAEL in healthy subjects as 6000 mg/day. Most of the studies that they targeted humans were reports such as dose-finding trials to reveal the nutritional requirements and the effectiveness of L-Lys-enriched food (wheat), since L-Lys is an essential amino acid. Meanwhile, they identified few intervention trials involving comparisons with a control group, such as randomized control trials. Comparison with a control group is generally preferable when attempting to accurately characterize the occurrence of adverse events associated with an intervention. In addition, when performing a safety assessment by SR, we specify the maximum-available no-observed adverse effect from by comprehensively collecting the results of different research. Therefore, the NOAEL we have reported is not the value estimated statistically and we think that the value is nearly the same as the value called the observed safety level (OSL) (Hathcock and Shao 2008). Shao reported that the OSL for L-Arg is 20 g/day (Shao and Hathcock 2008). However, we think that an additional approach is needed to confirm this reported dosage as the reliable safe upper limit.

The hockey stick model is one of the response models used in pursuit of the threshold for the expression of toxicity in toxicological studies (Johnson et al. 2009). This model is characterized by a biphasic pattern with one area reflecting no dose response and the other area reflecting a dose response. We possibly find the true threshold using this model due to the true threshold which toxicity is expressed is considered to be in between NOAEL and LOAEL.

We proposed a change-point regression model (CPRM) as an analytical method that includes the above-mentioned threshold in it. We also reported the usefulness of weighted CPRM (w-CPRM) weighted by the heterogeneity between clinical studies used in an SR added to this model (Kuramochi and Hayamizu 2023). CPRM has been applied to estimate the required amounts of essential amino acids and proteins (Hayamizu et al. 2011, 2021), but to the best of our knowledge no examples of its application to assessing the safety of amino acids in SR have been reported.

In this study, we performed an SR including only randomized double-blind controlled trials as intervention trials, given that they feature a control group, and conducted an assessment of the safety of L-Arg intake in healthy people using the occurrence of gastrointestinal symptoms as an index. Specifically, we applied w-CPRM and considered NOAEL based on the results of 31 studies (including 35 tests) as targets of SR.

## Methods

We conducted an assessment of the safety of L-Arg intake targeting healthy people by SR. We set the eligibility criteria of the study (PICOS) as follows: patients (P), healthy people who took L-Arg orally; intervention (I), L-Arg; comparison (C), placebo; outcome (O), any adverse events; and study design (S), intervention trial (randomized double-blind controlled trial).

We followed the Cochrane Handbook for Systematic Reviews of Interventions in conducting this SR and meta-analysis (Higgins and Green 2008). The results are reported in accordance with the PRISMA 2020 statement: updated guidelines for reporting systematic reviews (Page et al. 2021). The review protocol was registered at umin.ac.jp as UMIN000046133 before the beginning of the study.

### Study selection

A systematic search was performed using the PubMed, Cochrane Library, EBSCOhost, and Ichushi-Web databases to identify reports on studies involving L-Arg intervention in humans published until May 2021. Search terms included “L-Arg,” “double-blind,” and “randomized controlled trial” (Supplementary Table S1). To investigate all adverse events observed during the intervention trial, we included all oral L-Arg intervention studies without placing restrictions regarding background factors, environment, and sample size. Manual searches of journal articles and reference lists from relevant publications were performed to ensure that all appropriate studies were considered for inclusion. Unoriginal studies and duplicates were removed. Two investigators (KH and MM) performed the electronic search independently. Papers were chosen by title and contents of the abstract in the first screening, after which the full text was read. Then, papers describing studies in which L-Arg intervention was performed were selected in the second screening and finally those that matched the PICOS criteria were adopted.

### Inclusion and exclusion criteria

Studies identified from the systematic search were included or excluded according to the following criteria. The inclusion criteria were as follows: (1) study on healthy humans, (2) L-Arg administered orally, (3) L-Arg or L-Arg HCl forms as the intervention samples, and (4) double-blind randomized controlled trial. The exclusion criteria were as follows: (1) study design other than an L-Arg intervention study, (2) non-oral administration route of L-Arg, (3) L-Arg was not administered alone, (4) non-healthy humans, (5) L-Arg used as a salt for other acidic drugs, (6) meta-analysis, and (7) no information about the L-Arg dose.

### Data extraction

Two investigators (YK and MM) independently extracted the following data from eligible papers: (1) name of the first author, (2) year of publication, (3) study location, (4) study design, (5) numbers of participants in the L-Arg and control groups, (6) participant age, (7) L-Arg dosage per one-time and day, (8) duration of administration of L-Arg, and (9) AEs during the period of L-Arg treatment. Regarding the dosage, when L-Arg was used for intervention as a hydrochloride, it was converted to net L-Arg content. When information was ambiguous or missing, we contacted the corresponding author to obtain the most accurate data available.

### Methodological quality

Assessment of the quality of studies was conducted using the Cochrane Collaboration tool for assessing the risk of bias (RoB) (Higgins and Green 2008) and Jadad score (Jadad et al. 1996). The Cochrane Collaboration tool for assessing the risk of bias includes random sequence generation, allocation concealment, blinding of participants and personnel, blinding of outcome assessment, incomplete outcome data, selective reporting, and other sources of bias. Studies were classified as having high, low, or unclear risk of bias, according to each criterion.

Jadad score reflects five issues: was the study described as randomized, the randomization was adequate or not, was the study described as double-blind, the double blinding was adequate or not, and was there a description of withdrawals and drop outs. Jadad score was obtained by adding these results based on answers of “yes” =  + 1 and “no” = 0 (max. score 5). A score of ≥ 3 was considered to reflect relatively high quality.

### Classification of included papers

The included papers were categorized into the following three categories: Category A, with no descriptions of AEs in the article; Category B, stating that no AEs occurred; and Category C, mentioning that AEs occurred during the trial (Hayamizu et al. 2019).

### Estimation of NOAEL (Kuramochi and Hayamizu 2023)

The exploration of the threshold of L-Arg dosage for estimating NOAEL was conducted with weighted CPRM (w-CPRM) to take into account the heterogeneity of each study, which is one of the problems specific to SR. w-CPRM is defined as follows:

$${\omega }_{i}{y}_{i}={\omega }_{i}\mathrm{\beta {\rm I}}\left({\chi }_{i}>{\chi }_{cp}\right)\left({\chi }_{i}-{\chi }_{cp}\right)+{\omega }_{i}{\varepsilon }_{i}$$

where *y*_*i*_ is the risk difference (RD) and* x*_*i*_ is the dosage of L-Arg*.*$I$($\bullet$) = 1 if *x*_*i*_ > *x*_*cp*_ and is 0 otherwise. In this study, the weight of each study’s RD in the random effects model is ω_1*i*_, which is included in the formula for w-CPRM as shown below:

1 $${\omega }_{1i}{y}_{i}={\omega }_{1i}\mathrm{\beta {\rm I}}\left({\chi }_{i}>{\chi }_{cp}\right)\left({\chi }_{i}-{\chi }_{cp}\right)+{\omega }_{1i}{\varepsilon }_{i}$$

ω_1*i*_ is calculated as follows:

$${\omega }_{1i}=\frac{1}{{SE}_{i}^{2}+\widehat{{\tau }^{2}}}$$

Here, SE_*i*_ is the standard error of RD of each study, while τ^2^ denotes variability among the true effects that sample characteristics may introduce (Viechtbauer and Wolfgang 2010).

We considered true NOAEL using w-CPRM including the above-mentioned weight (${\omega }_{1i}$). Comparison of each model and setting of the dosage threshold were conducted by the maximum likelihood method used Akaike’s information criterion (AIC) as an indicator (Akaike 1992).

Safety was assessed using the frequency of AEs in the L-Arg and control groups. The variable modified RD and 95% CIs were further used to calculate pooled risk estimates. Cochran’s *Q* tests and I^2^ statistics were used to examine heterogeneity between studies. The random effects model was used for data synthesis. Sensitivity analysis was performed to identify any study responsible for heterogeneity and/or to test the validity of the conclusions by omitting one study sequentially (leave-one-out approach). Publication bias or small study effect was assessed by the funnel plot method and using Egger’s test (Egger et al. 1997). The meta-analysis and summary of the risk of bias were conducted using Cochrane Program Review Manager (RevMan) version 5.4 (The Cochrane Collaboration 2020). Statistical analysis was conducted using R4.1.1 (The R Foundation for Statistical Computing 2021) with the packages “meta” and “metafor” (Schwarzer et al. 2015; Viechtbauer and Wolfgang 2010).

### Certainty of evidence

We assessed the certainty of evidence according to the Grading of Recommendations Assessment, Development and Evaluation (GRADE). This was based on four grades, namely high quality, moderate quality, low quality, and very low quality, for the following five items: study limitations, inconsistency of results, indirectness of evidence, imprecision, and reporting bias (Guyatt et al. 2008).

## Results

**Fig. 1:**
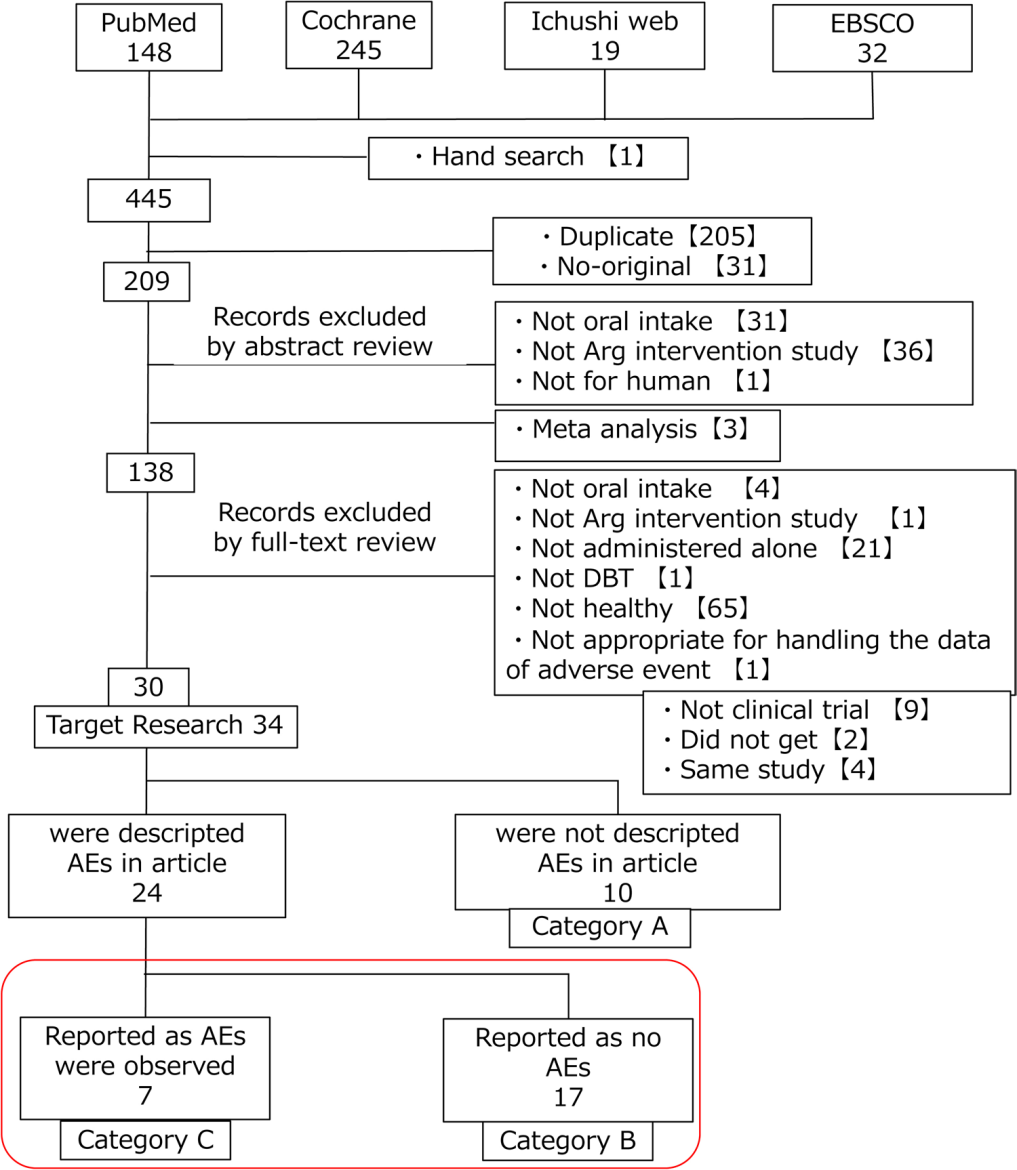
Flow chart of the selection of studies for inclusion in the systematic review. Articles in Category A did not describe AEs. Articles in Category B described AEs but reported no-observed AEs. Articles in Category C reported the occurrence of AEs. *AE* adverse event, *DBT* double-blind trial

Of the 445 papers retrieved through electronic and manual searches, 307 papers were excluded after screening of the title and abstract; then, 108 papers were excluded after full-text review. Thus, 34 studies (30 papers) met the inclusion criteria of the review protocol (Fig. 1). These 34 target studies were classified into Categories A–C. Category A included articles not describing the presence or absence of AEs (10 studies). Category B included articles reporting that no occurrence of an AE was observed (17 studies). Category C included articles reporting that the occurrence of an AE was observed (7 studies). The study by McNeal et al. (2018) was excluded because they intervened only subjects who were tolerable against for L-Arg and we regarded handling of the AE data was not appropriate.

### Characteristics of included studies

The characteristics of the included studies classified into Categories B and C are summarized in Table 1. For the research containing the results of two or more studies reported in the same paper, we listed the results separately. The proportion of men ranged from 0 to 100%, with a mean age of the subjects in each study was 10–73.8 years. The dosage of L-Arg tested in the studies ranged from 2000 to 30,000 mg/day (or/one-time dose), and the duration of administration ranged from 1 to 84 days.

**Table 1:** Characteristics of the included studies in systematic review

Study	Country	*n*_Arg_/*n*_total_	Age (year)	Inclusion criteria	Daily dose (mg)	One-time dose (mg)	Duration of trial (day)	Jadad score	Category
Adams et al. (1995)	Australia	12/12	34 ± 1	Healthy	21,000	7000	3	4	C
Aguiar et al. (2016)	Brazil	10/20	71.6 ± 5.9	Healthy	8000	8000	1	4	B
Alvares et al. (2012a, b)	Brazil	ND/15	arg:26.3 ± 4.9, pla:24.7 ± 1.8 (mean ± SD)	Healthy	4961.5	4961.5	1	4	B
Alvares et al. (2012a, b)	Brazil	9/17	26 ± 4.6 (mean ± SD)	Healthy	4961.5	4961.5	1	3	A
Alvares et al. (2014)	Brazil	8/15	arg:36.8 ± 7.1, pla:30.6 ± 9.5	Healthy	4947.8	1236.95	28	5	A
Andrade et al. (2018)	Brazil	10/20	22.8 ± 3.4	Healthy	6000	6000	3	3	B
Ast et al. (2011)	Poland	7/19	37.9 ± 8.03 (mean ± SD)	Healthy	6000	2000	28	3	B
Ast et al. (2011)	Poland	6/19	37.9 ± 8.03 (mean ± SD)	Healthy	12,000	4000	28	3	B
Birol et al. (2019)	Turkey	10/19	18.3 ± 0.48 (mean ± SD)	Healthy	10,800	10,800	1	3	A
Blum et al. (2000a, b)	The USA	10/10	55 ± 5	Postmenopausal healthy women	9000	3000	30	3	B
Bode-Böger et al. (1998)	Germany	8/8	25.2 ± 0.2	Healthy	6000	6000	1	3	A
Bode-Böger et al. (2003)	Germany	12/12	73.8	Healthy	16,000	8000	14	3	C
Chin-Dusting et al. (1996)	Australia	8/16	arg:21.9 ± 0.6, Pla:20.9 ± 1.0 (mean ± SEM)	Healthy	20,000	10,000	28	4	B
Fahs et al. (2009)	The USA	18/18	24.2 ± 0.7 (mean ± SE)	Healthy	7000	7000	1	4	A
Forbes and Bell (2011)	Canada	14/14	25 ± 5	Healthy	5850	5850	1	3	B
Forbes and Bell (2011)	Canada	14/14	25 ± 5	Healthy	11,700	11,700	1	3	C
Forbes et al. (2013)	Canada	15/15	28 ± 5 (mean ± SD)	Healthy	5850	5850	1	2	B
Forbes et al. (2014)	Canada	14/14	25 ± 4 (means ± SD)	Healthy	6150	6150	1	4	C
Fricke et al. (2008)	Germany	15/30	arg:54.4 ± 4.1, pla:55.3 ± 4.4	Healthy	14,200	ND	180	3	A
Luiking et al. (1998)	The Netherlands	10/10	24.2 ± 4.1	Healthy	30,000	7500	8	4	B
Mansoor et al. (2005)	Canada	7/7	37.4	Healthy	9923	3307.67	2	4	A
Meirelles and Matsuura (2018)	Brazil	12/12	27 ± 3 (mean ± SD)	Healthy	4961.5	4961.5	1	3	B
Pahlavani et al. (2014)	Iran	28/56	20.85 ± 4.29	Healthy	2000	2000	45	4	C
Robinson et al. (2003)	The UK	6/6	25 ± 2	Healthy	10,000	10,000	1	3	C
Savoye et al. (2006)	France	8/8	36	Healthy	15,000	15,000	1	4	B
Savoye et al. (2006)	France	8/8	36	Healthy	30,000	30,000	1	4	C
Schwedhelm et al. (2008)	Germany	20/20	57(NR)	Healthy	3000	1000	7	2	A
Schwedhelm et al. (2008)	Germany	20/20	57(NR)	Healthy	3200	1600	7	2	A
Streeter et al. (2019)	The USA	30/30	20.4 ± 1.8	Healthy	3000	3000	1	4	A
Suzuki et al. (2017)	Japan	10/42	20–49	Healthy	2000	2000	7	4	B
Ueno et al. (2018)	Japan	15/30	10–17	Healthy	5000	2500	84	4	B
Vanhatalo et al. (2013)	The UK	18/18	22 ± 3 (mean ± SD)	Healthy	6000	6000	1	4	B
Vignini et al. (2010)	Italy	40/80	AN:22 ± 5, healthy:23 ± 3	AN, healthy	8300	ND	14	3	B
Vuletic et al. (2013)	Croatia	59/117	21.7 ± 1.8	healthy	3000	3000	1	4	B

*ND* no data, *AN* anorexia nervosa

### Description of study quality

**Fig. 2:**
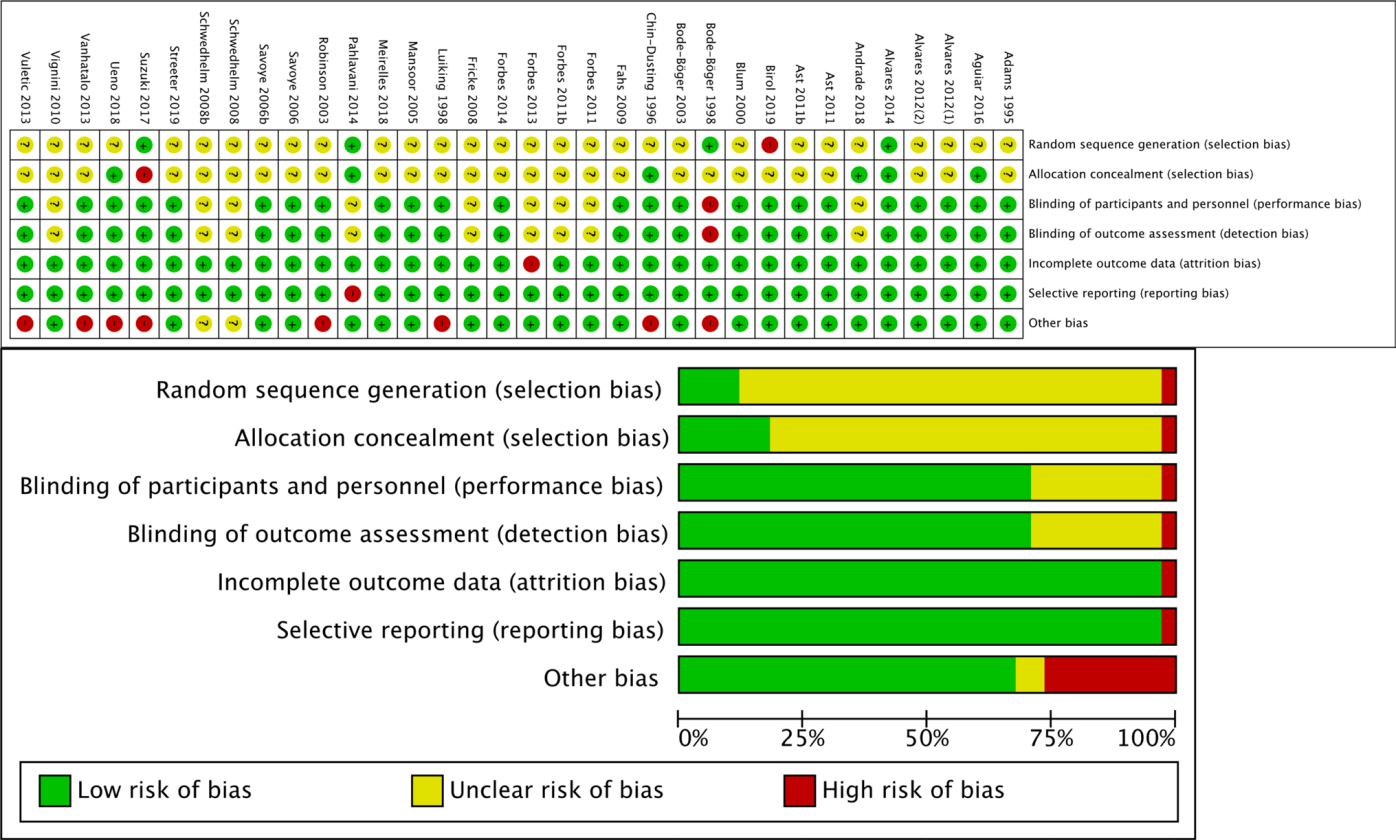
Assessment of risk of bias for 30 selected human studies: summary of items of bias. Risk of bias for all trials is presented as percentages of trials with low, high, or unclear risk of bias in each assessment item

The included studies varied in terms of their quality (Fig. 2). In all target study, only one in each item except of "other bias" was evaluated as high risk of bias and others were evaluated low or unclear risk of bias. Nine studies were evaluated high risk of bias to "other bias". In most cases, the high risk of bias was due to support, such as supplies and funds, being provided by a company. Other reasons for high risk of bias included the exclusion of results from time points in which it was considered that L-Arg could not be effective and there being an insufficient wash-out period in a cross-over trial. In terms of the Jadad score, 3 studies had a score of ≤ 2, while 31 studies had a score of ≥ 3. In addition, among the 24 studies corresponding to Categories B and C, all studies were of high quality with a score of ≥ 3, with one exception in which the study had a score of 2 (Table 1).

### Maximum dose, duration of administration, and sample size

**Fig. 3:**
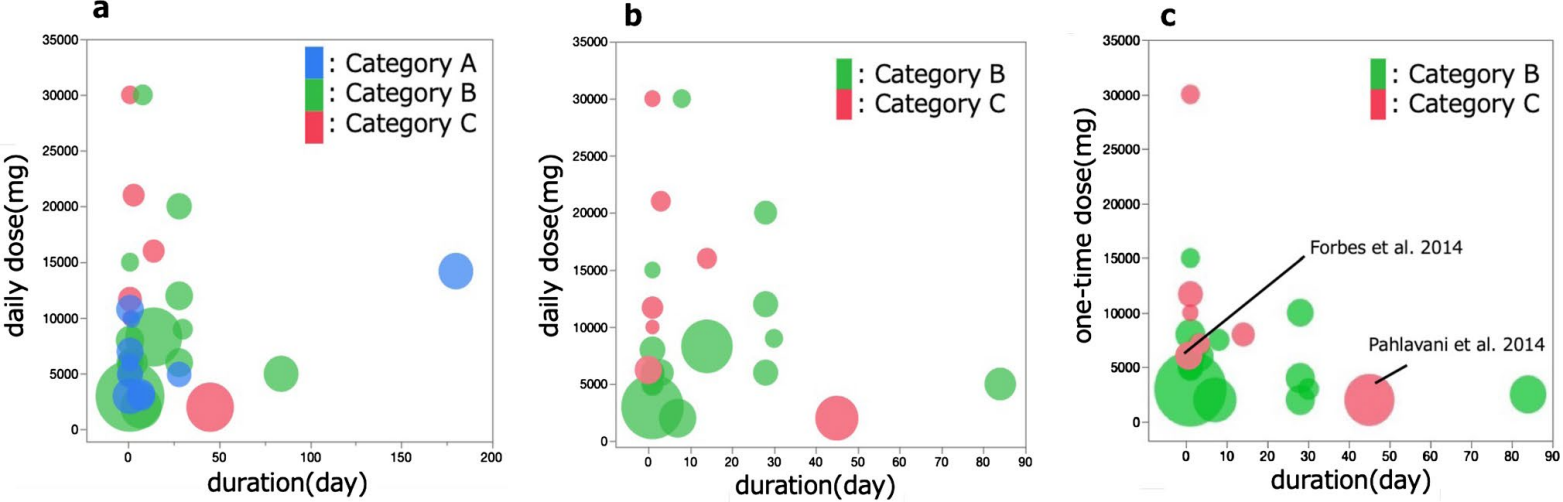
Summary of all included studies. The x- and y-axes indicate the duration of administration and the dosage of L-Arg, respectively. The size of the bubble indicates the sample size of the study. **Panel a:** all included studies, **panel b:** Categories B and C, which are needed to decide NOAEL and LOAEL, **panel c:** the dose of Categories B and C shown as mg/ one-time dose

Figure 3 provides a summary of the studies included in this SR as bubble plots. The x- and y-axes indicate the duration of administration and the dosage of L-Arg, respectively. The size of the bubble indicates the sample size of the study. Figure 3-a shows a breakdown of the 34 studies targeted for evaluation. Meanwhile, Fig. 3-b shows the 24 studies classified into Categories B and C in which the occurrence or absence of AEs could be judged. We set the primary endpoint as gastrointestinal symptoms because these have been reported most among the AEs. Gastrointestinal symptoms were defined as nausea, vomiting, diarrhea, and abdominal problems, such as cramps and bloating. We decided that it is crucial to consider the use of one-time dose because gastrointestinal symptoms are considered a relatively acute response. Therefore, we showed L-Arg dose per day and per one-time, respectively. The x-axis in Fig. 3-a and -b indicates the daily dose of L-Arg, while that in Fig. 3-c indicates the one-time dose. Figure 3-c shows the breakdown of 23 studies with the exception of that by Vignini et al. (2010) because it did not report the dosage per one-time administration. Among the studies in Category A, the highest dose was 14,200 mg/person/day (there was no description of how many doses L-Arg was divided into) and the longest duration of administration was 180 days (both the highest and the longest doses were administered by Fricke et al. 2008). Meanwhile, the largest sample size was 30 (Fricke et al. 2008; Streeter et al. 2019). Reports on studies within Category A did not describe the presence or absence of AEs; therefore, the information obtained from Category A was the longest dosing period and dose of L-Arg in humans as an amount added to an ordinary diet.

In studies within Category B, the highest dose and the longest duration of administration were 30,000 mg/day (Luiking et al. 1998) and 84 days (Ueno et al. 2018), respectively. The highest one-time dose was 10,000 mg (20,000 mg as a daily dose) (Chin-Dusting et al. 1996).

In Category C, the highest dose and the longest duration of administration were 30,000 mg/day (Savoye et al. 2006) and 45 days (Pahlavani et al. 2014), respectively. The highest one-time dose was 30,000 mg (Savoye et al. 2006).

Since the reports on the studies within Categories B and C described the presence and absence of AEs, we could use them to estimate the safety of L-Arg. From the Category B and C data, the maximum dose of L-Arg with no AEs was 30,000 mg/person/day as an amount added to an ordinary diet. Administration was performed for 8 days and the L-Arg dosage was divided up into four administrations (7500 mg/person/ one-time dose) (Luiking et al. 1998). Meanwhile, the minimum dose at which an AE apparently caused by L-Arg was observed was 2000 mg/person/day in a single dose (Pahlavani et al. 2014). However, the RD of the rate of occurrence of gastrointestinal symptoms was 0.04 (95% CI: − 0.06–0.13), which was not statistically significant (*P* = 0.45). Therefore, 6105 mg/person/day in a single dose, which was the second highest reported dose within Category C, became the observed LOAEL (o-LOAEL) (Forbes et al. 2014). For that reason, o-NOAEL was estimated at 6000 mg/person/ one-time dose. We estimated the NOAEL of a daily dose as 12,000 mg/day (4000 mg t.i.d), which is under the o-NOAEL and is the highest dose in Category B (Ast et al. 2011).

### Meta-analysis of AEs

**Fig. 4:**
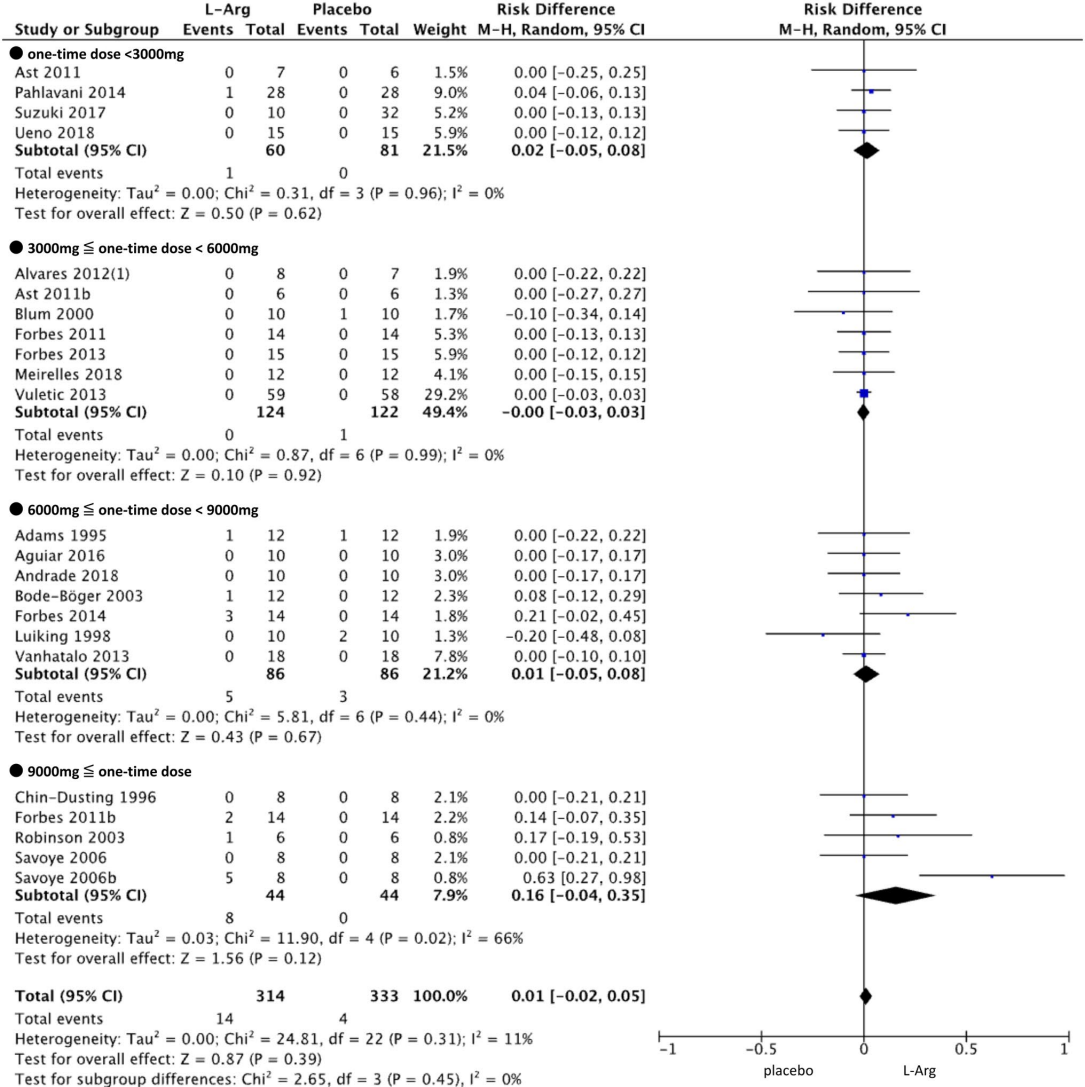
Difference in risk of suffering gastrointestinal symptoms associated with L-Arg. The included gastrointestinal symptoms were nausea, vomiting, diarrhea, and abdominal problems, such as cramp and bloating. Meta-analysis was carried out by stratified analysis based on the four different dose ranges

**Fig. 5:**
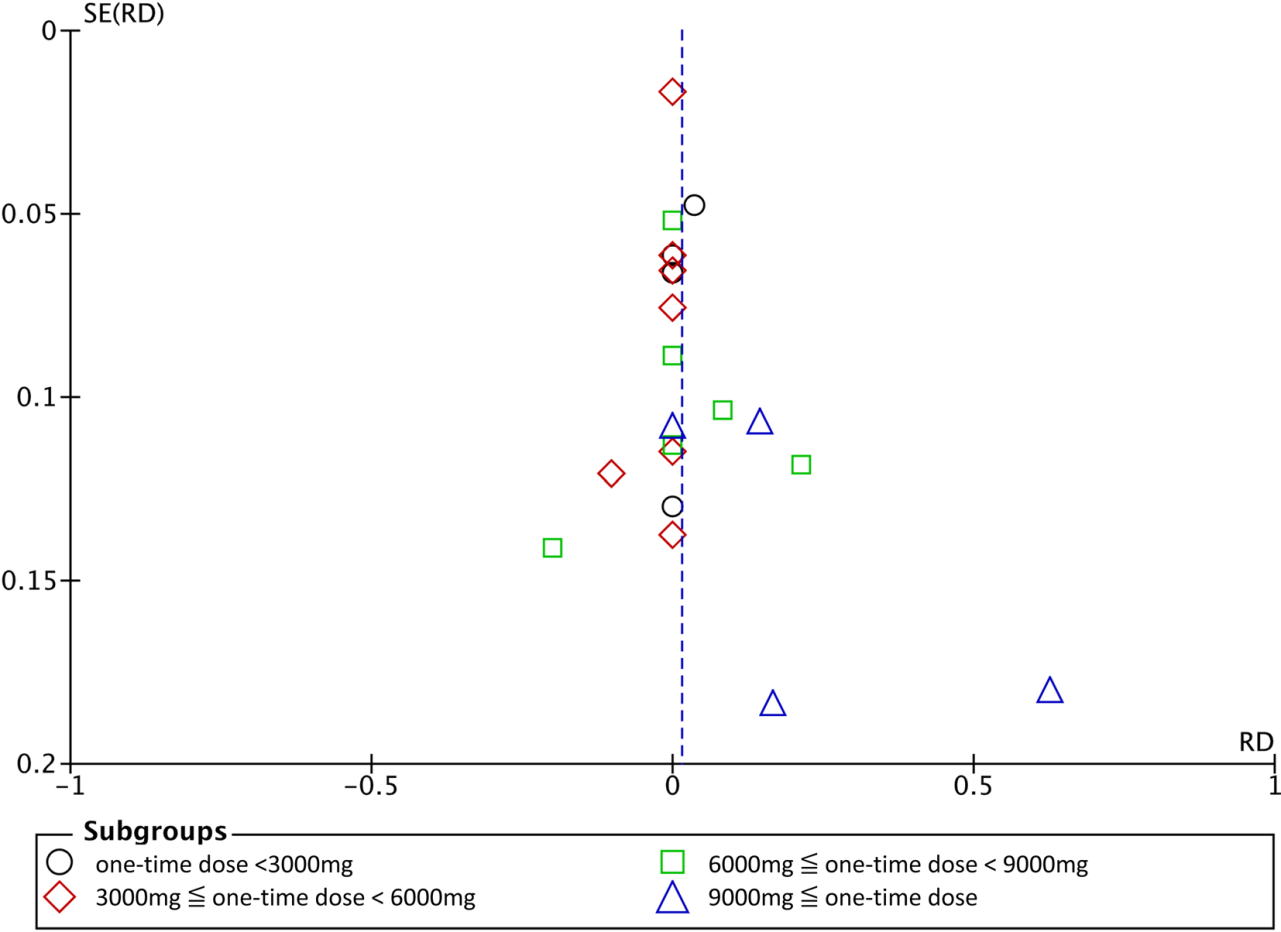
Results of funnel plot for L-Arg study. There was no evidence of publication bias from the funnel plot or Egger’s test (*P* = 0.1676)

We conducted a meta-analysis on the risk of AEs associated with L-Arg administration using the studies classified within Categories B and C (Fig. 4). The study by Vignini et al. (2010) was excluded because it did not report the amount of one-time L-Arg dose; thus, 23 studies were a target for the meta-analysis within Categories B and C. RD regarding the occurrence of gastrointestinal symptoms reported by Savoye et al. (2006) was 0.63 (95% CI: 0.27–0.98), which was significant (*P* = 0.0005). However, upon integrating all of the targeted studies, RD was 0.01 (95% CI: − 0.02–0.04), indicating no significant increase in gastrointestinal symptoms in association with L-Arg (*P* = 0.34). The heterogeneity among the studies was also small (*I*^2^ = 9%). Besides, we separated one-time dose into four levels (< 3000 mg,  ≤ 3000 to < 6000 mg,  ≤ 6000 to > 9000 mg, and ≥ 9000 mg), and considered the dose response by stratified analysis. RDs were 0.02 (95% CI: − 0.05–0.08), 0.00 (95% CI: − 0.03–0.03), 0.01 (95% CI: − 0.05–0.08), and 0.16 (95% CI: − 0.04–0.35), respectively, revealing a weak dose response. However, there were no significant differences in each subgroup. From the leave-one-out approach that was conducted for sensitivity analysis, slight changes in both *χ*2 value and *I*2 value were observed, but there were not seriously impact comparing to the overall effect. Therefore, there were no studies that were sufficiently heterogeneous to influence the integration result (Supplementary Table S2). In addition, no evidence of publication bias was identified from the funnel plot (Fig. 5) or Egger’s test (*P* = 0.1676).

### Estimation of NOAEL by w-CPRM

There are sometimes no threshold exists on the expression of AE depending on a kind of chemical. For that reason, we verified whether the threshold exists on the expression of AE to find NOAEL of L-Arg at first. The feasibility of applying CPRM was confirmed by comparing it to a linear regression model with AIC as an index. Here, Models A and B were established as linear regression models with no weighting and weighting by ω_1*i*_, along with Models C and D as CPRM (w-CPRM) with no weighting and weighting by ω_1*i*_, respectively.

Equations 2–4 are shown below:

2 $${y}_{i}={\beta \chi }_{i}+{\varepsilon }_{i}$$

3 $${\omega }_{1i}{y}_{i}={{\omega }_{1i}\beta \chi }_{i}+{\omega }_{1i}{\varepsilon }_{i}$$

4 $${y}_{i}=\mathrm{\beta {\rm I}}\left({\chi }_{i}>{\chi }_{cp}\right)\left({\chi }_{i}-{\chi }_{cp}\right)+{\varepsilon }_{i}$$

1 $${\omega }_{1i}{y}_{i}={\omega }_{1i}\mathrm{\beta {\rm I}}\left({\chi }_{i}>{\chi }_{cp}\right)\left({\chi }_{i}-{\chi }_{cp}\right)+{\omega }_{1i}{\varepsilon }_{i}$$

**Fig. 6:**
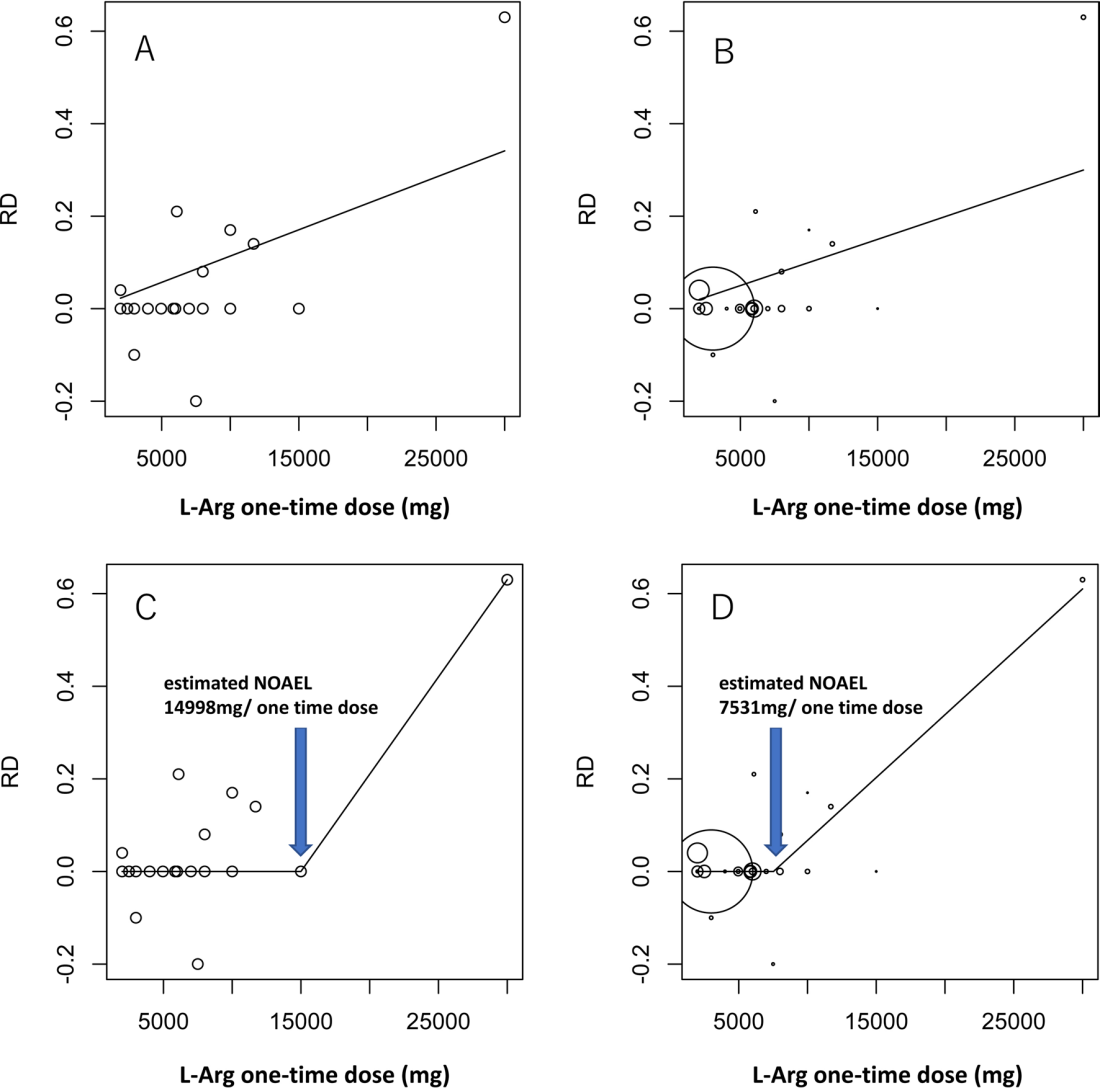
Results of regression model and change-point regression model (CPRM) for L-Arg study. Panels A and B indicate the results of Eqs. 2 and 3, respectively. Panels C and D indicate the results of Eqs. 1 and 4, respectively. The sizes of the circles in panels B and D indicate weight 1. Panels A and C are drawn by an unweighted model, so the sizes of the circles are the same. *RD* risk difference

In Fig. 6, the x-axis represents L-Arg dose per one-time administration, while the y-axis is the RD between the L-Arg group and the placebo group regarding the occurrence of gastrointestinal symptoms. Here, the size of the bubble reflects the weighting. (The sizes of the bubbles of Model A and Model C are certain because there is no weighting.)

In all of Models A to D, these results were statistically significant and the occurrence of AEs was recognized to exhibit a dose–response relationship (Table 2). Next, we recognized that there was a threshold in the dose response regarding the occurrence of AEs because Models C and D fitted better than Models A and B, as shown by comparing their AICs. Model D representing w-CPRM was the best fitting among the four models. Therefore, the estimated NOAEL by this model was calculated to be 7531 mg/one-time dose.

**Table 2:** Summary of models

Model	Weight	X_cp_	AIC_min_	Slope1	Slope2	*P*-value
A (*Eq. *2)	–	–	-31.14	1.137e-05	–	0.000236
B (*Eq. *3)	${\omega }_{1}$	–	-38.62	9.991e-06	–	0.000463
C (*Eq. *4)	–	14,998	-46.38	0	4.200e-05	1.33e-07
D (*Eq. *1)	${\omega }_{1}$	7531	-62.31	0	2.716e-05	4.26e-09

### Certainty of evidence

We confirmed variation in the certainty of evidence from the assessment of the RoB of the 23 studies targeted for the meta-analysis. Random sequence generation and allocation concealment asked to report appropriately due to focus on the existence of AEs in this research. In addition, "other bias" out of the result of RoB stands out for high risk of bias compares with other items but the most of reasons were funding from companies related to materials of L-Arg. The gastrointestinal symptoms that we focus on in this work are considered relatively temporary or tolerable. Therefore, in some reports, it is described that the intervention continued until the end of the study even if AEs occurred. As such, it is easy to believe that the gastrointestinal AEs were minor events. In addition, we thought that there were few reports related to AEs among studies targeted here because the researchers had emphasized assessing the effectiveness rather than the safety in view of the fact that L-Arg is one of food components considered safe. For that reason, we thought that there is a serious risk of bias in the outcome that only used studies gathered this time and we judged that it is appropriate to get one level of grade down according to GRADE.

From the result of the meta-analysis about the frequency of gastrointestinal symptoms, *I*^2^ was small as 11% (*P* = 0.31) and we also confirmed the CIs between each study overlap. In the study by Savoye et al., five subjects had gastrointestinal symptoms among eight were reported and we judged that there was a significant difference in the result. Generally, *I*^2^ can be considered to reflect low heterogeneity among studies when it is 40% or less. However, from the results of the leave-one-out analysis, no individual study has an influence that changes the whole outcome of this research. Therefore, we judged that there is no problem with “inconsistency of results” according to GRADE.

In terms of the inclusion criteria applied in this study, the research by Blum et al. (2000a, b) targeted postmenopausal women but healthy. Meanwhile, research by Vignini et al. (2010) included subjects with anorexia nervosa (AN) besides healthy subjects, but we selected it here as a target study for inclusion in this research. However, we did not use the data from Vignini et al. in the meta-analysis and w-CPRM because there was no report the amounts of the one-time dose applied. In addition, Savoye et al. (2006) administered L-Arg in the stomach through a gastric tube, but this is considered equivalent to oral intake. In this work, we also included studies in which L-Arg intervention involved L-Arg as a hydrochloride, not only L-Arg alone. Nonetheless, we thought there were no differences between them because the main substances themselves were both L-Arg. In terms of the establishment of a control group, outcome, and study design, all studies met the inclusion criteria and there were no points worthy of special mention. Therefore, all studies were considered to adhere to the PICOS guidelines that we set and we judged that there were no problems with “indirectness of evidence” according to GRADE.

Generally, when conducting meta-analyses, a total number of events of 300 or more is preferable in the case where the outcome is a binary variable. However, in this meta-analysis regarding the risk of gastrointestinal symptoms being associated with L-Arg administration, the number of events is below 300 and the 95% confidence interval of RD is included 0. Therefore, it was very serious about "imprecision" and was equivalent to two levels of grade down according to the criteria of GRADE. However, GRADE was devised the assessment of effectiveness of guidelines and so on. Our research is focused on the safety, for that reason, we thought some issues remine to use it directly. Here, NOAEL was calculated as 7531 mg/one-time dose by w-CPRM. Therefore, we conducted meta-analysis with all studies that used an L-Arg dose of 7531 mg/one-time dose or more, and we observed a trend of an increasing incidence of AEs (*P* = 0.06). When the outcome of a study is AEs, caution is needed even if a tendency for a significant difference is identified. In a study like the current one, we might not obtain a sufficient total number of events even if we achieve a large sample size because the rate of AEs due to food components is considered low. Therefore, in this research, we considered that it was not particularly essential to fulfill the criteria "reporting bias" according to GRADE.

In terms of the reporting bias, the results of the funnel plot and Egger’s test confirmed that there was no serious influence of such bias. From the assessment of the five items: study limitations, inconsistency of results, indirectness of evidence, imprecision and reporting bias, we eventually judged the certainty of the evidence as being intermediate by considering that there weren’t enough reasons that don’t judge no problems about “study limitations” and “imprecision”.

## Discussion

In this study, we conducted an assessment of the safety of the oral intake of L-Arg based on studies targeting healthy people using SR. The estimation of NOAEL was conducted by w-CPRM, which assumed that the incidence of adverse events until reaching the threshold of the expression is 0 and modified the intercept (Kuramochi and Hayamizu 2023).

In our study, gastrointestinal symptoms were the most frequently reported as adverse events, therefore, we conducted by focusing on the occurrence of these events. Previous studies have also reported gastrointestinal symptoms, such as nausea and diarrhea, as adverse events with L-Arg ingestion (Holeček 2022; Barbul 1986; Evans et al. 2004). Adverse events other than gastrointestinal symptoms among the target studies included headache in 2 cases and skin problems in 3 cases (Supplementary Table S3). The subject who had a bullous pemphigoid was withdrawn from the study due to the necessity of steroid treatment. However, there was no description of causal relationships with L-Arg (Blum et al. 2000a, b). All patients out of the five cases mentioned above dropped out except one case of headache, but there were no other reports of serious adverse events including deaths or hospitalizations. Most of the participants who had gastrointestinal symptoms continued participating until the study ended because these were mild or temporary. No laboratory findings were obtained from the target studies. The study by McNeal was excluded from the target studies of our SR because it was limited only to healthy subjects who tolerated high-dose L-Arg. However, there were no effects on the clinical laboratory data between 0 and 90 days nevertheless the dosage was relatively high: 15000 mg/one-time dose (30000 mg/day) for 90 days (McNeal et al. 2018).

Among the 34 studies that were targeted for analysis, from the results of meta-analysis on the studies classified into Categories B and C, which reported the existence of AEs, there was no significant association between the reported gastrointestinal symptoms and the intake of L-Arg. There were also no significant results in each group from the subgroup analyses for each singly administered dose category. However, we observed a trend for a dose-dependent increase in RD. Therefore, the rate of occurrence of gastrointestinal AEs might rise upon increasing the one-time dosage of L-Arg, suggesting the need to decide on the upper limit of one-time administered dose.

In the conventional method that was used for the safety assessment of L-Lys, we decided on o-NOAEL from the studies classified into Categories B and C and then estimated NOAEL (Hayamizu et al. 2019). However, as mentioned in the introduction, this dose is the same as OSL (Hathcock and Shao 2008). Therefore, we estimated NOAEL using CPRM, which enables to find the exploration of the threshold, as a novel approach this time.

Most meta-analyses are based on sets of studies that are not exactly identical in terms of their methods and/or the characteristics of the included samples. Such differences may obscure the true effects (Viechtbauer and Wolfgang 2010). Meta-analyses generally use either the fixed effects model or the random effects model. The fixed effects model is a model that assumes the random errors to the difference between studies and the random effects model is a model that inserts the heterogeneity that can't be ignored the difference between studies to the fixed effects model. Therefore, meta-analyses use these errors and differences as weight (ω_1*i*_). For that reason, we considered that the same adjustment would also be needed on CPRM. Here, we used the random effects model considering the differences between studies, which is a problem specific to SR. However, τ2 that denotes variability among the true effects tat sample characteristics may introduce was 0.00 from the result of Fig. 4 (*τ*^2^ = 0.03 in the subgroup analysis at doses of 9000 mg or more), and it was interpreted that the heterogeneity among studies was low. Therefore, we thought that the same result could get when using the fixed effects model this time.

In this research, we adopted only RCT, which are generally considered to be the highest-quality study type and assessed the quality of studies. However, variations in both the score of the Cochrane Collaboration tool for assessing the risk of bias (Higgins and Green 2008) and the Jadad score (Jadad et al. 1996) were identified among the studies, suggesting that the studies were not of uniform quality (Tables 1 and 2). Meanwhile, the RoB results are not normally used as a parameter for meta-analysis. For this reason, we thought to reflect the result of RoB into the w-CPRM as weight to find the true NOAEL considering about the heterogeneity of it.

Here, we considered using the result for RoB from Cochrane Collaboration tool for assessing the risk of bias, which is widely used in SR, and integrating it as ω_2*i*_ into w-CPRM.

5 $${\omega }_{2i}*{y}_{i}={\omega }_{2i}*\mathrm{\beta {\rm I}}\left({\chi }_{i}>{\chi }_{cp}\right)\left({\chi }_{i}-{\chi }_{cp}\right)+{\omega }_{2i}*{\varepsilon }_{i}$$

The Cochrane assessment has seven items in total, so we regarded a perfect score as 14, with high quality of items as + 2, unclear as + 1, and low quality as 0. We regarded the total score of each study as score_i_ and we regarded RS_i_ as score_i_ divided by 14 (reflecting full marks), which we used to adjust ω_1*i*_.

$${\omega }_{2i}={\omega }_{1i}* {\mathrm{RS}}_{i}=\frac{1}{{\mathrm{SE}}_{i}^{2}+\widehat{{\tau }^{2}}}*{\mathrm{RS}}_{i}$$

$${\mathrm{RS}}_{i}=\frac{{\mathrm{score}}_{i}}{14}$$

Here, Model E is a linear regression model weighted by ω_2*i*_, while Model F is w-CPRM weighted by ω_2*i*_. The formula of the linear regression model weighted by ω_2*i*_ is:

6 $${\omega }_{2i}{y}_{2i}={{\omega }_{2i}\beta \chi }_{2i}+{\omega }_{2i}{\varepsilon }_{i}$$

Models E and F are represented by Eqs. 6 and 5, respectively. The AICs of Models E and F were − 38.01 and − 61.77, respectively (Table 3). This indicates that L-Arg has NOAEL when using ω_2*i*_ as well as ω_1*i*_. NOAEL weighted by ω_2*i*_ was calculated 8754 mg/ one-time dose. This was a higher dosage but the AIC was slightly bigger than the one weighted by ω_1*i*_, that meant the fitting of the model was worse.

**Table 3:** Summary of models with weight2

Model	Weight	X_cp_	AIC_min_	Slope1	Slope2	*P*-value
E (*Eq. *6)	${\omega }_{2}$	–	– 38.01	1.058e-05	–	0.000259
F (*Eq. *5)	${\omega }_{2}$	8754	– 61.77	0	2.912e-05	2.5e-09

When using CPRM, at least three data points on each of two straight lines and over seven data points as a whole are necessary to accurately estimate the dose. We could judge that sufficient data were obtained to apply CPRM because here the target research for the assessment involved 23 studies. However, when applying CPRM that has no weighting (Model C), the straight line after the dose threshold was limited to two points, with 30,000 mg/ one-time dose being the highest reported dose and 15,000 mg/ one-time dose being the next highest (Fig. 6). Therefore, there were fewer data points on the side of higher dosages, which limited the estimation of the dose. However, the result of the estimation of NOAEL using this model did not appear to have been markedly impacted because three or more data points were present on both straight lines in w-CPRM weighted by ω_1*i*_ and ω_2*i*_.

In this research, there were ten reports corresponding to Category A, which refers to studies with no description of the existence of AEs. We could not use these reports for the meta-analysis and w-CPRM, despite them being target studies for the assessment. As a reason of why these reports in category A exist, foods are generally considered safe. It also seems that one of the reasons why objective assessment of gastrointestinal symptoms is difficult is that there are less severe than other AEs and cannot be detected by a blood test. Therefore, we thought that there is a limit to estimating NOAEL using only data from SR.

When the L-Arg dose was 4000 mg/one-time dose, AIC was − 52.83 from the AIC plot of w-CPRM weighted by ω_1*i*_. This was the dose estimated using our conventional method to estimate NOAEL (OSL). AIC was − 62.31 when NOAEL was estimated as 7531 mg/one-time dose which was considered to approach the true value. It was also the same when using w-CPRM weighted by ω_2*i*_.

However, regarding ω_2*i*_, which includes the result of RoB, the estimated dose may vary widely depends on the kinds of RoB and how to insert them to weight. Therefore, we consider that there is a need for additional research about these issues.

## Conclusion

We evaluated the safety of L-Arg added to an ordinary diet through a systematic review of human studies. We judged that the NOAEL of L-Arg is 7531 mg/one-time dose from the result of w-CPRM using ω_1*i*_, which is the weighting typically used in meta-analyses.

### Supplementary Information

Below is the link to the electronic supplementary material.

Supplementary file1 (DOCX 13 KB)

Supplementary file2 (DOCX 23 KB)

Supplementary file3 (DOCX 19 KB)
