# Supplementary material for: Safety assessment of L-Arg oral intake in healthy subjects: a systematic review of randomized control trials
Source: Amino Acids. 2023 Nov 10;55(12):1949–64. doi: 10.1007/s00726-023-03354-6 (PMC10724322; doi:10.1007/s00726-023-03354-6)
Supplement: Supplementary file 1 — Supplementary file1 (DOCX 13 KB) [file 726_2023_3354_MOESM1_ESM.docx]

Supplementary Table S1 Search terms

| **Web databases** | **Search terms** |
| --- | --- |
| PubMed | L-Arginine [Ti] +double-blind (filters) |
| Cochrane Library | L-Arginine [Ti]+ double-blind |
| Ichushi-Web | L-Arginine [Ti] or アルギニン [Ti] (原著＋ランダム＋ヒト) |
| EBSCOhost | L-Arginine [Ti]+double-blind +randomized controlled trials |
