# Supplementary material for: Safety assessment of L-Arg oral intake in healthy subjects: a systematic review of randomized control trials
Source: Amino Acids. 2023 Nov 10;55(12):1949–64. doi: 10.1007/s00726-023-03354-6 (PMC10724322; doi:10.1007/s00726-023-03354-6)
Supplement: Supplementary file 2 — Supplementary file2 (DOCX 23 KB) [file 726_2023_3354_MOESM2_ESM.docx]

Supplementary Table S2 Leave-one-out sensitivity and heterogeneity analyses for L-arginine

|  | Arg group (n) | Control  group (n) | Quantitative data synthesis | | | |  | Heterogeneity analysis | | | |
| --- | --- | --- | --- | --- | --- | --- | --- | --- | --- | --- | --- |
|  |  |  | overall effect size | 95%Cl | Z value | P value |  | Tau^2^ | Chi^2^ | df(Q) | *I*^2^ |
| Overall effect | 314 | 333 | 0.01 | -0.02 : 0.05 | 0.87 | 0.39 |  | 0.00 | 24.81 | 22 | 11% |
| Leave-one-out sensitivity analysis |  |  |  |  |  |  |  |  |  |  |  |
| Ast 2011 | 307 | 327 | 0.02 | -0.02 : 0.05 | 0.89 | 0.37 |  | 0.00 | 24.95 | 21 | 16% |
| Pahlavani 2014 | 286 | 305 | 0.01 | -0.02 : 0.05 | 0.73 | 0.47 |  | 0.00 | 24.68 | 21 | 15% |
| suzuki 2017 | 304 | 301 | 0.02 | -0.02 : 0.05 | 0.91 | 0.36 |  | 0.00 | 25.06 | 21 | 16% |
| ueno 2018 | 299 | 318 | 0.02 | -0.02 : 0.05 | 0.92 | 0.36 |  | 0.00 | 25.01 | 21 | 16% |
| Alvares 2012(1) | 306 | 326 | 0.02 | -0.02 : 0.05 | 0.89 | 0.37 |  | 0.00 | 24.96 | 21 | 16% |
| Ast 2011b | 308 | 327 | 0.02 | -0.02 : 0.05 | 0.89 | 0.37 |  | 0.00 | 24.94 | 21 | 16% |
| Blum 2000 | 304 | 323 | 0.02 | -0.02 : 0.05 | 1.00 | 0.32 |  | 0.00 | 24.97 | 21 | 16% |
| Forbes 2011 | 300 | 319 | 0.02 | -0.02 : 0.05 | 0.91 | 0.36 |  | 0.00 | 25.01 | 21 | 16% |
| Forbes 2013 | 299 | 318 | 0.02 | -0.02 : 0.05 | 0.92 | 0.36 |  | 0.00 | 25.01 | 21 | 16% |
| Meirelles 2018 | 302 | 321 | 0.02 | -0.02 : 0.05 | 0.91 | 0.36 |  | 0.00 | 25.01 | 21 | 16% |
| Vuletic 2013 | 255 | 275 | 0.02 | -0.02 : 0.05 | 1.04 | 0.3 |  | 0.00 | 21.98 | 21 | 4% |
| Adams 1995 | 302 | 321 | 0.02 | -0.02 : 0.05 | 0.90 | 0.37 |  | 0.00 | 25.11 | 21 | 16% |
| Aguiar 2016 | 304 | 323 | 0.02 | -0.02 : 0.05 | 0.90 | 0.37 |  | 0.00 | 24.99 | 21 | 16% |
| Andrade 2018 | 304 | 323 | 0.02 | -0.02 : 0.05 | 0.90 | 0.37 |  | 0.00 | 24.99 | 21 | 16% |
| Bode-Boger 2003 | 302 | 321 | 0.01 | -0.02 : 0.04 | 0.76 | 0.45 |  | 0.00 | 23.95 | 21 | 12% |
| Forbes 2014 | 300 | 319 | 0.01 | -0.02 : 0.03 | 0.54 | 0.59 |  | 0.00 | 20.14 | 21 | 0% |
| Luiking 1998 | 304 | 323 | 0.02 | -0.02 : 0.05 | 1.05 | 0.29 |  | 0.00 | 24.59 | 21 | 15% |
| Vanhatalo 2013 | 296 | 315 | 0.02 | -0.02 : 0.05 | 0.93 | 0.35 |  | 0.00 | 24.99 | 21 | 16% |
| Chin-Dusting 1996 | 306 | 325 | 0.02 | -0.02 : 0.05 | 0.90 | 0.37 |  | 0.00 | 24.97 | 21 | 16% |
| Forbes 2011b | 300 | 319 | 0.01 | -0.02 : 0.04 | 0.65 | 0.52 |  | 0.00 | 22.24 | 21 | 6% |
| Robinson 2003 | 308 | 327 | 0.01 | -0.02 : 0.04 | 0.79 | 0.43 |  | 0.00 | 23.50 | 21 | 11% |
| Savoye 2006 | 306 | 325 | 0.02 | -0.02 : 0.05 | 0.90 | 0.37 |  | 0.00 | 24.97 | 21 | 16% |
| Savoye 2006b | 306 | 325 | 0.01 | -0.02 : 0.03 | 0.48 | 0.63 |  | 0.00 | 10.24 | 21 | 0% |
