# Supplementary material for: Safety assessment of L-Arg oral intake in healthy subjects: a systematic review of randomized control trials
Source: Amino Acids. 2023 Nov 10;55(12):1949–64. doi: 10.1007/s00726-023-03354-6 (PMC10724322; doi:10.1007/s00726-023-03354-6)
Supplement: Supplementary file 3 — Supplementary file3 (DOCX 19 KB) [file 726_2023_3354_MOESM3_ESM.docx]

Supplementary Table S3 Details of the adverse events

| Study | Adverse events | | Daily dose  (mg) | One-time dose  (mg) | Duration of trial  (day) | Category |
| --- | --- | --- | --- | --- | --- | --- |
|  | L-Arginine | placebo |  |  |  |  |
| Adams et al. 1995 | Abdominal bloating (n=1)  Mild headaches (n=1) | Abdominal bloating (n=1) | 21000 | 7000 | 3 | C |
| Blum et al. 2000 | Bullous pemphigoid (n=1: drop out) | Abdominal pain (n=1: drop out) | 9000 | 3000 | 30 | B |
| Bode-Böger et al. 2003 | Diarrhea (n=1) | - | 16000 | 8000 | 14 | C |
| Forbes et al. 2011b | Mild GI distress (n=2) | - | 11700 | 11700 | 1 | C |
| Forbes et al. 2014 | Vomited (n=3: drop out)  Light headache (n=1: drop out) | - | 6150 | 6150 | 1 | C |
| Luiking et al. 1998 | - | Nausea (n=2) | 30000 | 7500 | 8 | B |
| Pahlavani et al. 2014 | Stomach problem (n=:1drop out)  Skin dermatitis (n=2: drop out) | - | 2000 | 2000 | 45 | C |
| Robinson et al. 2003 | Stomach discomfort (n=1) | - | 10000 | 10000 | 1 | C |
| Savoye et al. 2006b | Bloating and diarrhea (n=5) | - | 30000 | 30000 | 1 | C |
